# Interfacial Separations by a Polydimethylsiloxane Layer. Molecular Modeling of Coated Stir Bar Extraction of Organics from Aqueous Solutions

**DOI:** 10.1021/acs.langmuir.5c00113

**Published:** 2025-04-17

**Authors:** Abdulazez Alzhrani, Cynthia J. Jameson, Sohail Murad

**Affiliations:** † Department of Chemical and Biological Engineering, Illinois Institute of Technology, Chicago, Illinois 60616, United States; ‡ Department of Chemistry, University of Illinois at Chicago, Chicago, Illinois 60607, United States

## Abstract

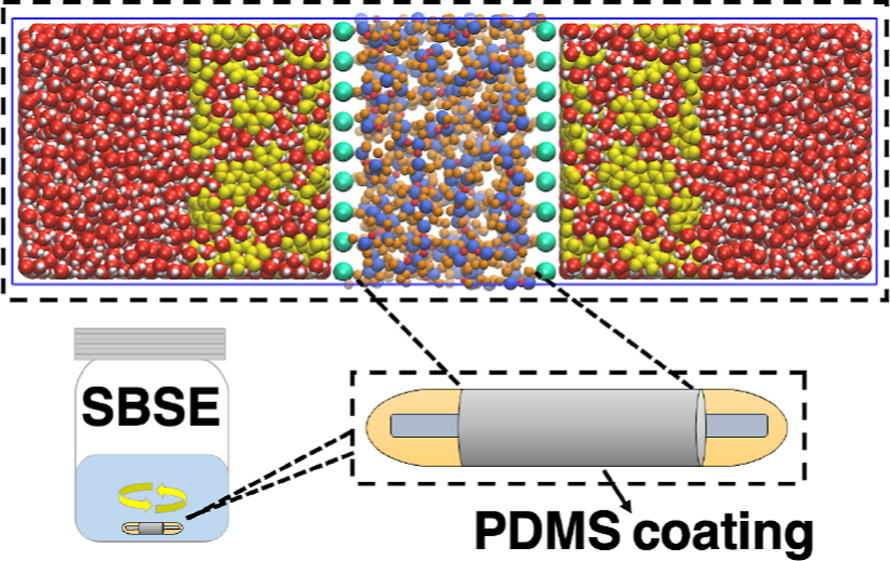

Separation processes relying on interfacial interactions, such as the stir bar sorptive extraction represent one of the most critical methods of analyte trace organic detection and extraction in environmental, food, and biomedical samples. While the use of polydimethylsiloxane (PDMS) as a sorptive coating in SBSE has exhibited high sensitivity and efficiency; the molecular mechanisms involved are less explored. We report molecular simulation studies using molecular dynamics (MD) to investigate the absorption of organic compounds including phenol, chlorophenol, guaiacol, benzyl alcohol, and phenethyl alcohol at the aqueous-PDMS interface, and focus on temperature-dependent behavior. By employing an appropriate force field for PDMS, organic compounds, and water, these simulations directly predict PDMS-water partition coefficients, log P [PDMS/water], diffusion coefficients, and solubilities in the PDMS phase without relying on octanol–water partitioning as a surrogate. An important result of the MD simulations in this work is our ability to predict the temperature dependence of the log p(PDMS/water). Results reveal a nonmonotonic temperature-dependent sorption trend for log P [PDMS/water] values. However, we find that with increasing temperature, the absolute number of organic molecules in the PDMS phase increases, driven by enhanced molecular diffusion and PDMS’s significant sorption capacity. The findings demonstrate that performing SBSE at elevated temperatures can enhance analyte uptake, improving the analytical sensitivity of trace level extractions, where achieving sufficient analyte concentration in the sorptive phase is critical for reliable detection and quantification in a wide variety of applications in environmental monitoring, food safety, and biomedical analysis. These simulations predict that temperature is a good parameter for the optimization of operating conditions of SBSE. Our results also highlight the ability of MD simulations to reliably capture complex molecular level interactions governing SBSE performance, aligning well with experimental trends and observed behaviors.

## Introduction

Separation processes that depend on interactions at the surface area that exists between two immiscible phases such as liquid–liquid or liquid–solid interfaces have been widely employed for extracting critically desired molecules or removing unwanted impurities. In particular, stir bar sorptive extraction (SBSE) uses a coated stir bar for extraction of organic analytes from aqueous solution. The coating material is typically polydimethylsiloxane (PDMS) at a film thickness of 0.5 mm consisting of a PDMS mixture that has a density of 0.825 g cm^–3^.^1^ The stir bar is dropped into the aqueous solution sample and while it stirs it absorbs and concentrates organic compounds into the sorbent coating. When the SBSE-step has been completed, the analytes are desorbed using thermal desorption and GC/MS determination of the concentrated organic compounds can be performed in one integrated system. In this work, we carry out molecular modeling of the process by which interactions at the aqueous-PDMS interface permit the organic compounds in the aqueous sample to be continuously absorbed into the PDMS layer. Estimates of the separation efficiency has traditionally been done by using octanol–water-partition coefficients of compounds as a surrogate for the actual PDMS-water partition coefficients. In this work, we are able to determine the latter by classical molecular dynamics simulations of the water-organic, PDMS-organic, and water-PDMS interactions at the interface between the liquid phases.

Environmental pollution is a widespread and increasing concern throughout the world, with noxious contaminants presenting significant hazards to both ecosystems and public health.^2,3^ Among organic pollutants, polycyclic aromatic hydrocarbons (PAHs), polychlorinated biphenyls (PCBs), phenolic compounds, and aromatic alcohols are of greater concern because of their persistence, toxicity, and recalcitrance to biodegradation.^4−7^

Among these, we consider phenolic compounds and benzyl alcohol derivatives.^4^ The control of these pollutants demands sensitive analytical methodology. Among conventional methods, liquid–liquid extraction (LLE) and solid phase extraction (SPE) have been in use in commercial laboratories,^2,8^ but they require multistep operations for extraction, purification, and concentration, hence are time-consuming as well as involve large volumes of organic solvents.^2,9^

Stir bar srptive extraction (SBSE) overcomes some of the drawbacks associated with traditional approaches. Introduced in the late 1990s by Baltussen et al.^1^ and subsequently commercialized under the name “Twisters”, SBSE simplifies sample preparation by exploiting a coated stir bar for trace analyte preconcentration with limited handling and at high sensitivity. Continuous stirring favors contact between analytes and the sorptive surface, thus enhancing the efficiency of extraction. After extraction, coupled detectors like mass spectrometry (MS) provide quantification with an enhanced detection limit with reduced matrix interference.^10^

Efficiency and selectivity of SBSE depends on many factors, particularly coating material. Polydimethylsiloxane (PDMS), chemically inert and thermally stable, provides effective extraction for a wide variety of both nonpolar and moderately polar analytes.^11−13^ It has been demonstrated that SBSE is very effective for environmental analysis. Concentrations of detected phenol range from 43 to 138 μg/L; detection limit was as low as 0.1 μg/L for 2,4-dichlorophenol.^14−16^ SBSE has also been used to address food safety and biomedical concerns.^17−20^ Comparative studies underline the supremacy of SBSE over the classical techniques such as LLE and SPE. For instance, it achieves much higher recoveries of PAHs in complex matrixes, recovering up to 288% more analytes than LLE.^21^

Temperature is another important operating variable that affects the performance of SBSE. The effect of temperature can vary depending on the chemical nature of the analyte, including its polarity, hydrophobicity, and functional groups. While higher temperatures increase the kinetics of extraction, they usually decrease the partition coefficients, hence limiting the overall efficiency. Therefore, temperature and extraction time should balance for best results.^22^

Whereas the advances in experimentation have significantly widened the applications of SBSE, understanding the mechanisms at the molecular level has not been a priority. This is where computational techniques, particularly molecular dynamics (MD) simulations, can offer a reliable way of bridging experimental gaps and looking deeper into the molecular processes of stir bar sorptive extraction. Control of extreme conditions, like high temperatures and pressures, is easily achievable in MD simulations, even for conditions which may be difficult to access experimentally. Simulations provide highly detailed spatial and temporal insight at a molecular level into the phenomena of analyte diffusion, partitioning, and interaction within the PDMS phase.

In this work, we are using a nonequilibrium molecular dynamics simulation methodology, providing further insight with variable temperature studies. Building upon previous research regarding the competitive adsorption of siloxanes and water onto silica surfaces as described by Floess and Murad, we apply similar modeling approaches in our studies of the behavior of organic molecules in the 80:20 mixture of the PDMS matrix.^23^ By using molecular simulations, it will be possible to determine the influence of temperature on the extraction efficiency. For any given application, simulations can predict whether high or low temperatures are likely to optimize the extraction. Molecular level understanding is crucial for improving the performance of SBSE on a wide range of compounds under various conditions. The MD simulations conducted in this work predict properties such as density, self-diffusion, and logP[PDMS/water] partition coefficient directly, without resorting to octanol as a surrogate for PDMS. Comparisons against available experimental trends show these MD simulations capture the trends well and hence offer a controlled environment to explore the molecular interactions and verify the effectiveness of SBSE for specific analytes, providing insight into molecular mechanisms influencing the performance of SBSE.

## Methodology

The force field used is shown below.
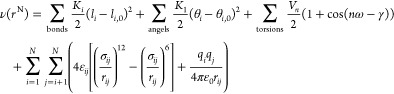
1where intramolecular interactions are expressed in the usual way with bond stretches, bond angle and dihedral angle terms, and nonbonded interactions are modeled by Lennard-Jones and Coulomb terms.^30^

### Refining and Validating the PDMS Model

Reliable force field models of PDMS, water, and organic molecules are necessary in order to get quantitative results of partition coefficient, log *P* [PDMS/water]. We chose a currently available United Atom Model as a starting point.^24^ For our studies getting the liquid density of PDMS correct was critical to ensure that the PDMS had the right free volume and “cavities” for the partitioning studies to be conducted. We thus modified the parameters to ensure good agreement with the available experiment density of PDMS at a range of temperatures. The Lennard-Jones parameter σ of CH_3_ was changed by 3.49% to better capture the experimental trends of the measured densities of PDMS. The original potential was resulting in the density of the PDMS being 8% too high, which would seriously compromise our proposed studies. All our simulations in this study were performed using a 1 fs time step using the LAMMPS molecular dynamics software package.^25^

In the case of cross-interactions, Lorentz–Berthelot (L–B) mixing rules were initially applied. We would like to point that L–B mixing rules do not correctly represent cross interactions^26^ when the molecules are structurally different (often overestimating or underestimating them), and especially for properties that are very sensitive to such cross interactions such as solubilities.^27^ We found out as described later that the water–phenol interactions were not correctly represented by the L–B rules so they had to be adjusted by a factor of 1.2.

The short-range interactions were computed with an overall cutoff distance of 12 Å. Long-range electrostatic interactions were assessed by means of the particle–particle–particle–mesh (PPPM) solver.^28^ The combined use of intramolecular and nonbonded potentials can describe the PDMS system rather realistically provided the bonded and nonbonded interactions are accurately modeled. The Lennard-Jones potential parameters corresponding to PDMS used in this simulation are given in Table 1.

**Table 1:** 12-6 Lennard-Jones Potential Parameters of Polydimethylsiloxane (PDMS)

pair	ε (kcal/mol)	σ[Å]	reference
Si–Si	0.1310	4.29	(24)
Si–O	0.0772	3.94	(24)
Si–CH_3_	0.1596	3.83	(24)
O–O	0.0800	3.30	(24)
O–CH_3_	0.1247	3.38	(24)
CH_3_–CH_3_	0.1944	3.86	this work

**Figure 1:**
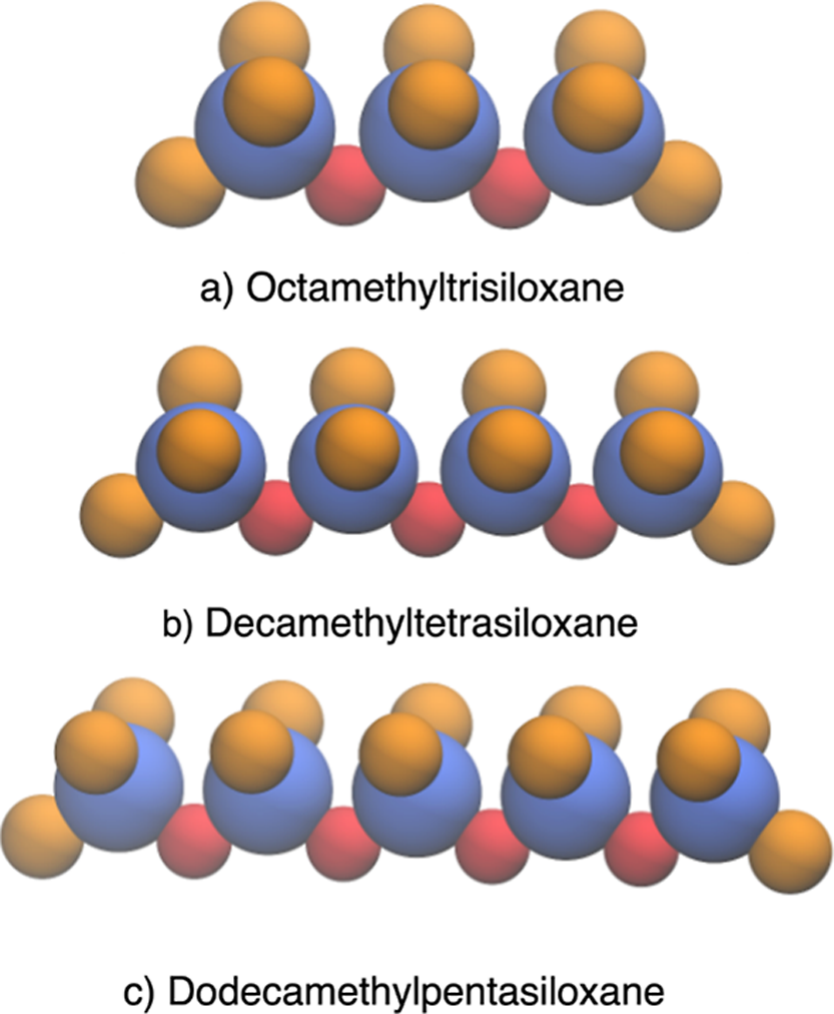
Polydimethylsiloxane (PDMS) oligomers with varying chain lengths, using blue for silicon (Si), red for oxygen (O), and orange for methyl groups (−CH_3_) attached to the silicon atoms. (a) PDMS (*n* = 1): octamethyltrisiloxane. (b) PDMS (*n* = 2): decamethyltetrasiloxane. (c) PDMS (*n* = 3): dodecamethylpentasiloxane.

After developing our PDMS potential model, we studied three oligomers of PDMS represented in Figure 1, showing the inherently flexible nature of the polymer due to repeating siloxane units (−Si–O–Si–), where each Si atom is bonded to two methyl groups (−CH_3_). Subsequently, developing these structure models involved system equilibration for appropriate initial conditions which could allow accurate description of average properties. PACKMOL was used in all simulations to generate nonoverlapping initial packing.^29^ The molecular visualizations were done using VMD (visual molecular dynamics software).^30^

**Figure 2:**
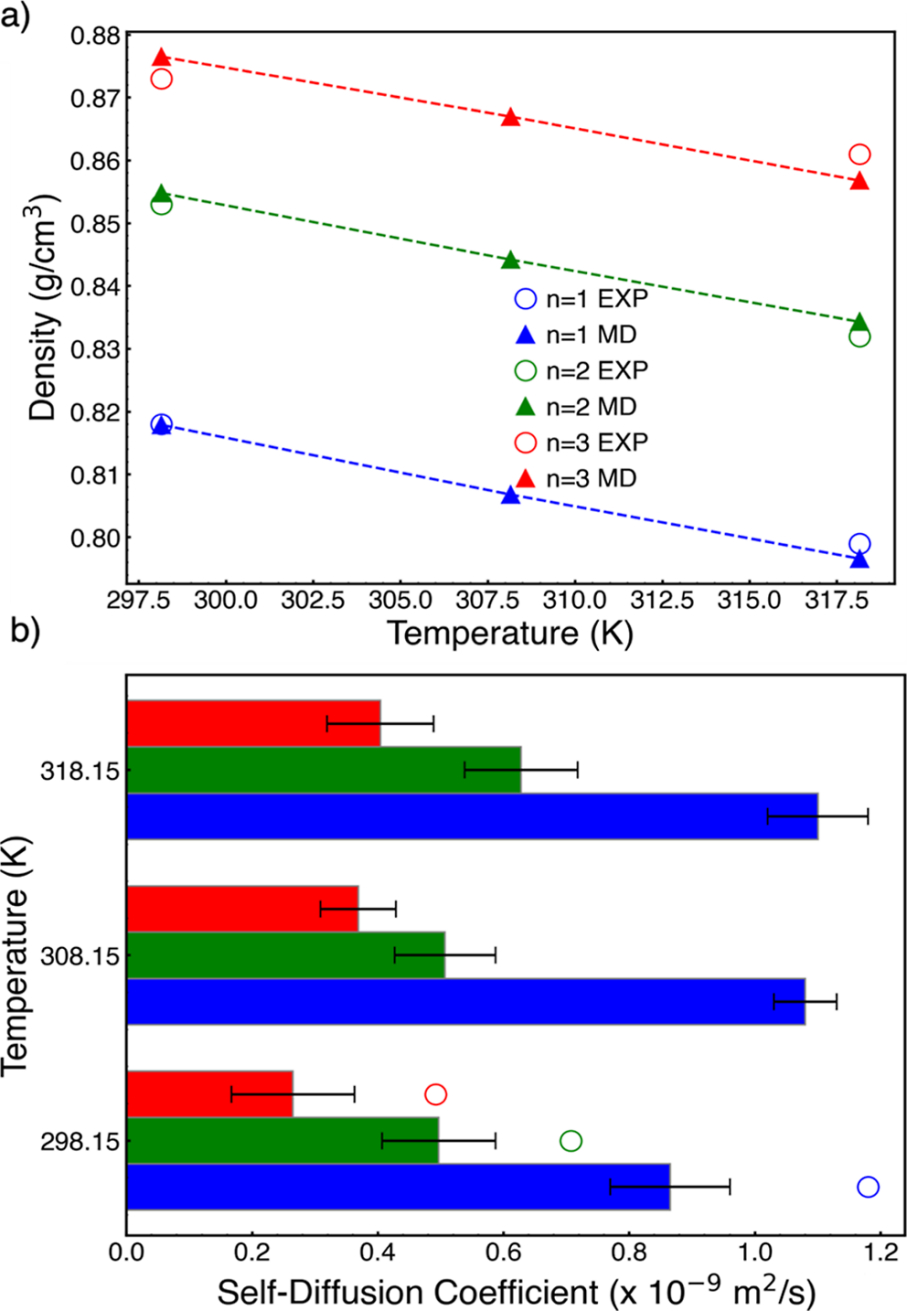
Temperature dependent properties of PDMS (*n* = 1, *n* = 2, *n* = 3). (a) Calculated density of PDMS (MD) compared with experiment (EXP); error bars lie within the symbols used for ata points. (b) Self-diffusion coefficients of PDMS: The *n* = 1 blue, *n* = 2 green, *n* = 3 red bars are from MD, this work. The experimental data at 298.15 K are shown as open circles of the same color, with unknown error bars.

**Figure 3:**
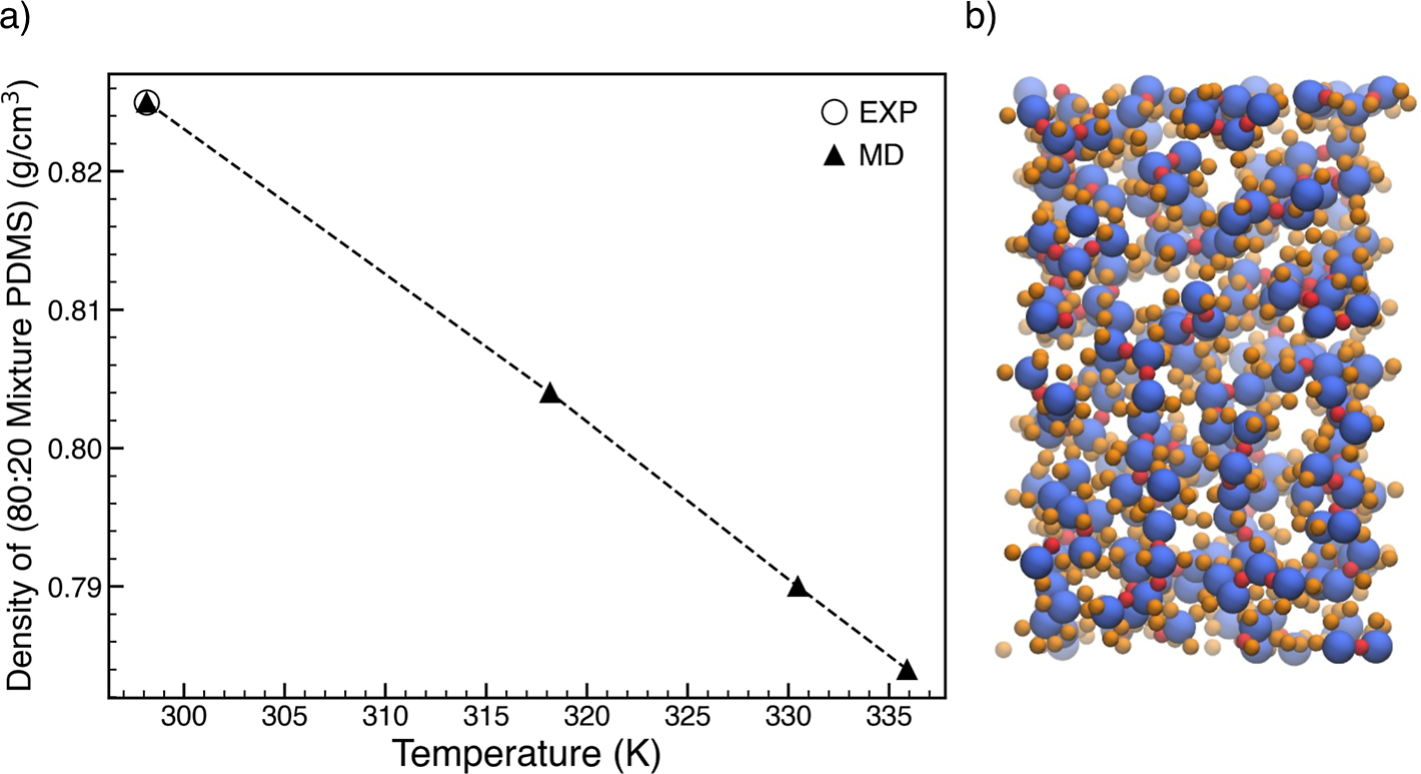
(a) The comparison of its simulated density with experiment, and (b) atomic configuration of 80:20 mixture of (*n* = 1: *n* = 2 PDMS). The error bars lie within the symbols used for data points.

Equilibration simulations were performed on all 3 types of PDMS oligomers (*n* = 1, 2, and 3) and the mixture 80:20 by weight of *n* = 1 and *n* = 2 respectively, which best matches the density 0.825 g cm^–3^ of the typical SBSE coating.^1^ This was done by performing annealing simulations in the *NPT* ensemble (constant numbers of particles, pressure, and temperature) for 10 ns over a range of temperatures. In all simulations, temperature and pressure were controlled via Nose–Hoover thermostat and barostat. The annealing simulations for each of three oligomers and the 80:20 mixture PDMS were conducted separately. Heating of the systems was carried out up to 408.15 K, followed by cooling to 298.15 K in steps of 10 K, equilibrated for 1 ns at each temperature to ensure stability. This allowed each system to approach equilibrium, as verified from the density and potential energy convergence as a function of time. After equilibration, we did a simulation in the *NVT* ensemble for another 5 ns in order to measure the diffusion coefficient and densities for the above PDMS systems, including the mixture. The results of MD simulations at various temperatures are shown by calculating densities Figure 2a and diffusion coefficients in Figure 2b for the individual oligomers. In Figure 3 we show a representation of the resulting configuration of the 80:20 mixture to be inserted into the simulation box, and its density as a function of temperature. As seen in these two figures, comparisons with experimental data^31,32^ show good agreement for the densities of the individual oligomers as a function of temperature which are the important properties for our application, and we capture the trend in the diffusion coefficients at 298.15 K. These constitute the validation of the modified force field for PDMS. The effective diffusion coefficients *D* are calculated through the Stokes–Einstein Law expressed by eq 2, where fits are performed linearly with the purpose of extracting the slope of MSD over time.
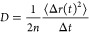
2Here, *n* = dimension number, and the ratio of MSD over the time interval is the slope from the plotted data.

### Validation of the Force Field for the Organic Molecules

**Figure 4:**
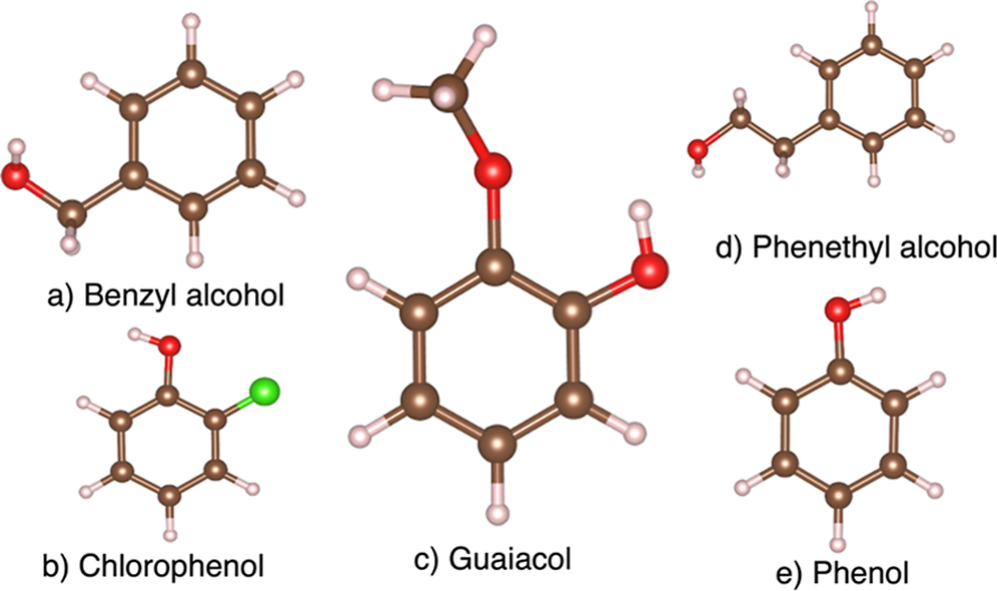
Structures of studied organic compounds. Hydrogen is represented by light balls, carbon is brown, oxygen is red and chlorine is green.

**Figure 5:**
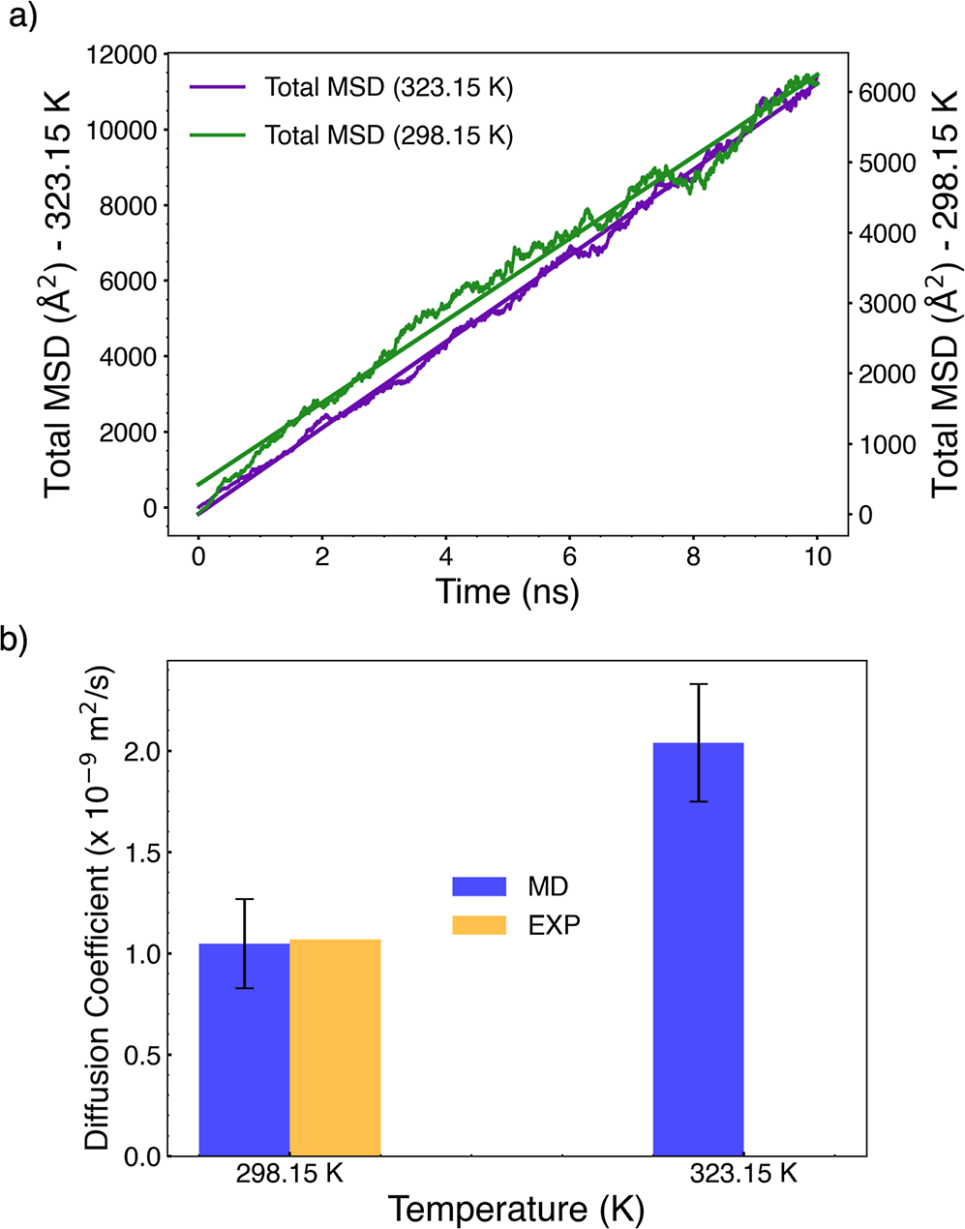
(a) MSD of phenol in water vs time at (298.15 and 323.15 K). Note the different scales on the *y* axis for data at the two temperatures. (b) Comparison of MD simulations and experimental results for diffusion coefficients of phenol in water.

Water was modeled using the SPC model, and bond lengths and angles were constrained using the SHAKE algorithm in order to maintain the rigidity of the water molecule.^33,34^ The organic molecules in Figure 4 were modeled using the OPLS-All Atom force field, starting with phenol for testing.^35^ Initial simulations gave a higher diffusion rate for phenol when compared with experimental results. To solve this disagreement and to obtain reliable force fields applicable to all organic molecules, we fitted the interaction parameters of water and phenol by increasing the epsilon of the cross term (the water–phenol interaction parameter) by a factor 1.20. (see earlier discussion of the L–B mixing rules in the section on potential models for PDMS) This modification, tested via measuring the diffusion coefficients of phenol in water, returned a highly consistent outcome in simulations versus the experimental diffusion rates, as reflected in Figure 5.^36^

The resulting refined parameters are used throughout for all organic molecules. This refinement is necessary to model the interactions and transport of phenol and, by extension, any organic species throughout this system. Applying this above validated setup, we extended the simulation methodology to a larger set of organic compounds, such as chlorophenol, guaiacol, benzyl alcohol, and phenethyl alcohol.

**Figure 6:**
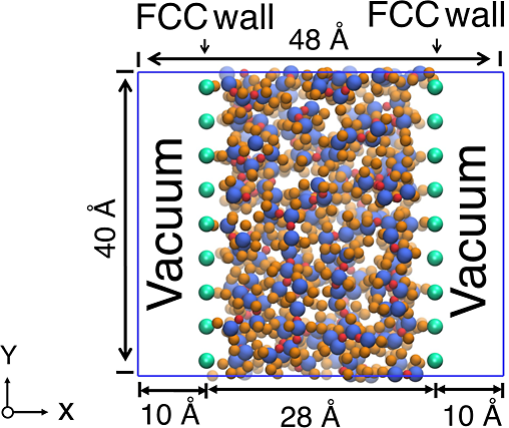
Simulation setup for annealing of PDMS system. The simulation box measures 48 Å × 40 Å × 40 Å and contains a 80:20 mixture by weight of *n* = 1 and *n* = 2 PDMS (28 Å thick) surrounded by vacuum layers (10 Å each) along the *x*-axis. FCC walls represented by green balls are applied at the boundaries to stabilize the system.

The simulation system was constructed featuring two face centered cubic (FCC) walls placed in the *x*-direction, setting the nonperiodic boundaries. The system is periodic, in the *y-* and *z*-directions. The FCC walls are rigid and prevent the motion of the PDMS phase in *x*-direction and are impermeable to PDMS but permits water and organic molecules to permeate. The design had vacuum gaps next to the walls at both ends and a middle section with the PDMS layer, ensuring the polymer chains remained aligned in the *x*-direction, as shown in Figure 6.

**Figure 7:**
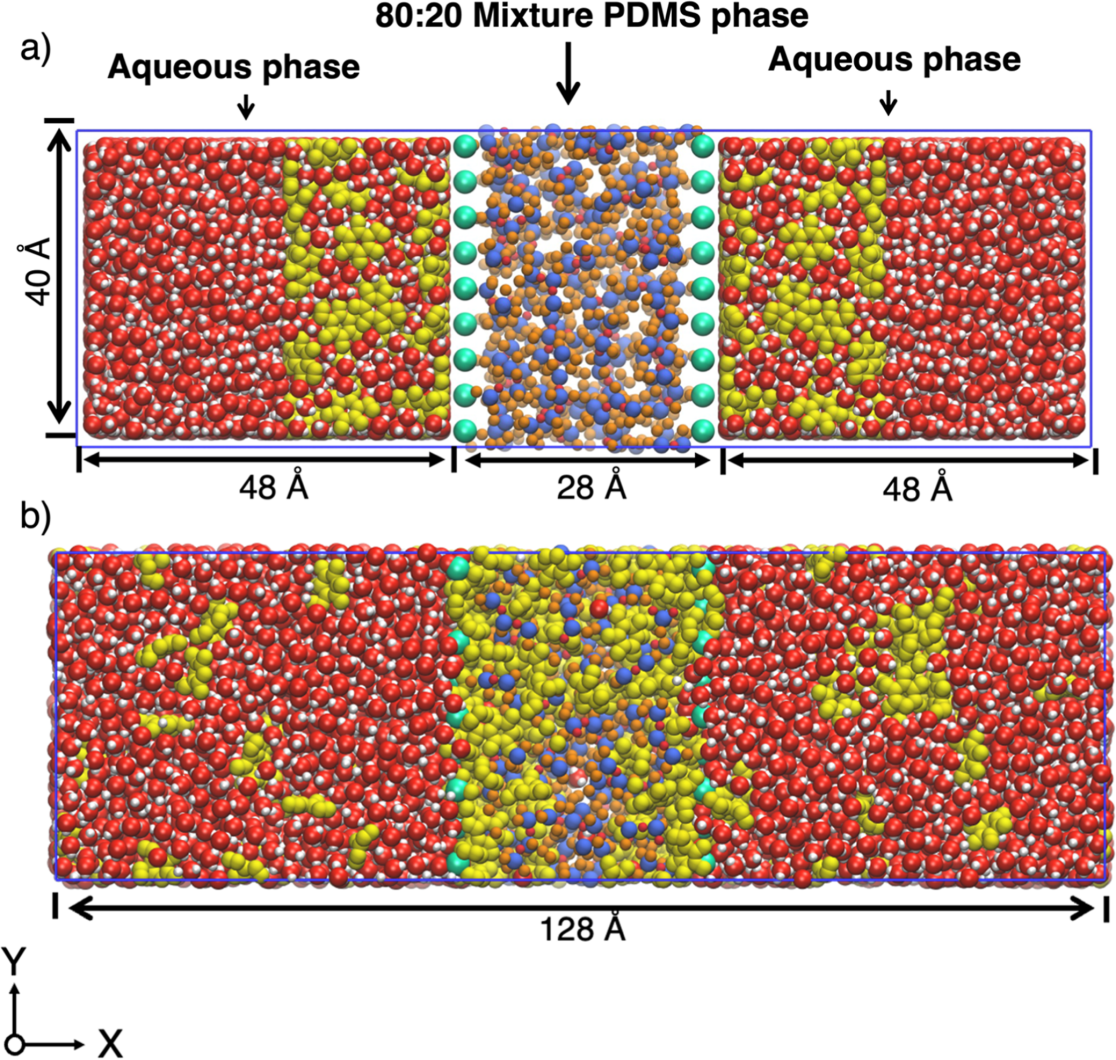
PDMS-Water System (a) initial system with 80:20 PDMS mixture (28 Å) between two aqueous phases (48 Å each). (b) System after 60 ns of simulation as an example. Actual simulation times are much longer than this.

After the equilibration of the 80:20 mixture PDMS slab, the aqueous phases were inserted, as seen in Figure 7a, a central PDMS phase of 80:20 mixture bounded by two aqueous phases. Such a setup allows PDMS to interface effectively with surrounding aqueous phases and allow the dynamics of organic compounds to be monitored.

The compounds in Figure 4 were intentionally chosen because of their desirable solubility limits in water, compatible with constraints imposed by the molecular dynamics simulations; that is, for statistically valid simulation results, we need to have a significant number of organic molecules in the aqueous phase for the size of the simulation box being used. They represent typical phenolic and aromatic compounds common in environment related studies, and thus are relevant objects for testing the performance of SBSE extraction. Solubility data for each compound obtained from literature are given in Table 2. All simulations involve only one type of organic molecule in order to simplify the analysis, and at experimental conditions.^20,22^ The maximum number of organic molecules placed in the aqueous phase was calculated according to the limit of its solubility in water.

**Table 2:** Solubility of Organic Compounds in Water

type of organics	MW (g/mol)	*T* (K)	solubility (mg/L)	solubility (mol %)	references
phenol	94.11	298.15	83,600	1.6	(37)
		315.15	-	-	-
		330.48	146,220	2.7	(40)
		335.89	190,210	3.5	(40)
guaiacol	124.14	298.15	18,700	0.3	(38)
chlorophenol	128.6	298.15	28,500	0.4	(39)
benzyl alcohol	108.14	298.15	42,900	0.71	(40)
phenethyl alcohol	122.16	298.15	22,200	0.3	(41)

The entire system contained 82 atoms forming the FCC walls, 89 PDMS molecules with 75 having *n* = 1 and 14 having *n* = 2, and 6418 water molecules. In each simulation, the number of organic molecules in the aqueous phase was adjusted based on their temperature-dependent solubility in water. Organic molecules were placed initially close to the PDMS phase for the purpose of bypassing the long simulation times for the organics to diffuse toward the PDMS layer. An initial saturated concentration of organic compound was used in the aqueous phase. The simulation was conducted in two stages. The first stage ensured the saturation of the PDMS phase, while the second stage involved measuring the partition coefficient log *P* [PDMS/water] Figure 7b shows a typical system configuration after 60 ns simulation as an example. For the computation of log P [PDMS/water], simulation times 65–100 ns were used and approach to equilibrium was verified in each case by plots like those in Figure 9, and the simulation times varied with temperature and organic compound. In addition, simulations were carried out in a box 140 Å × 40 Å × 40 Å to find that the results are statistically the same as for the 128 Å × 40 Å × 40Å box.

**Figure 8:**
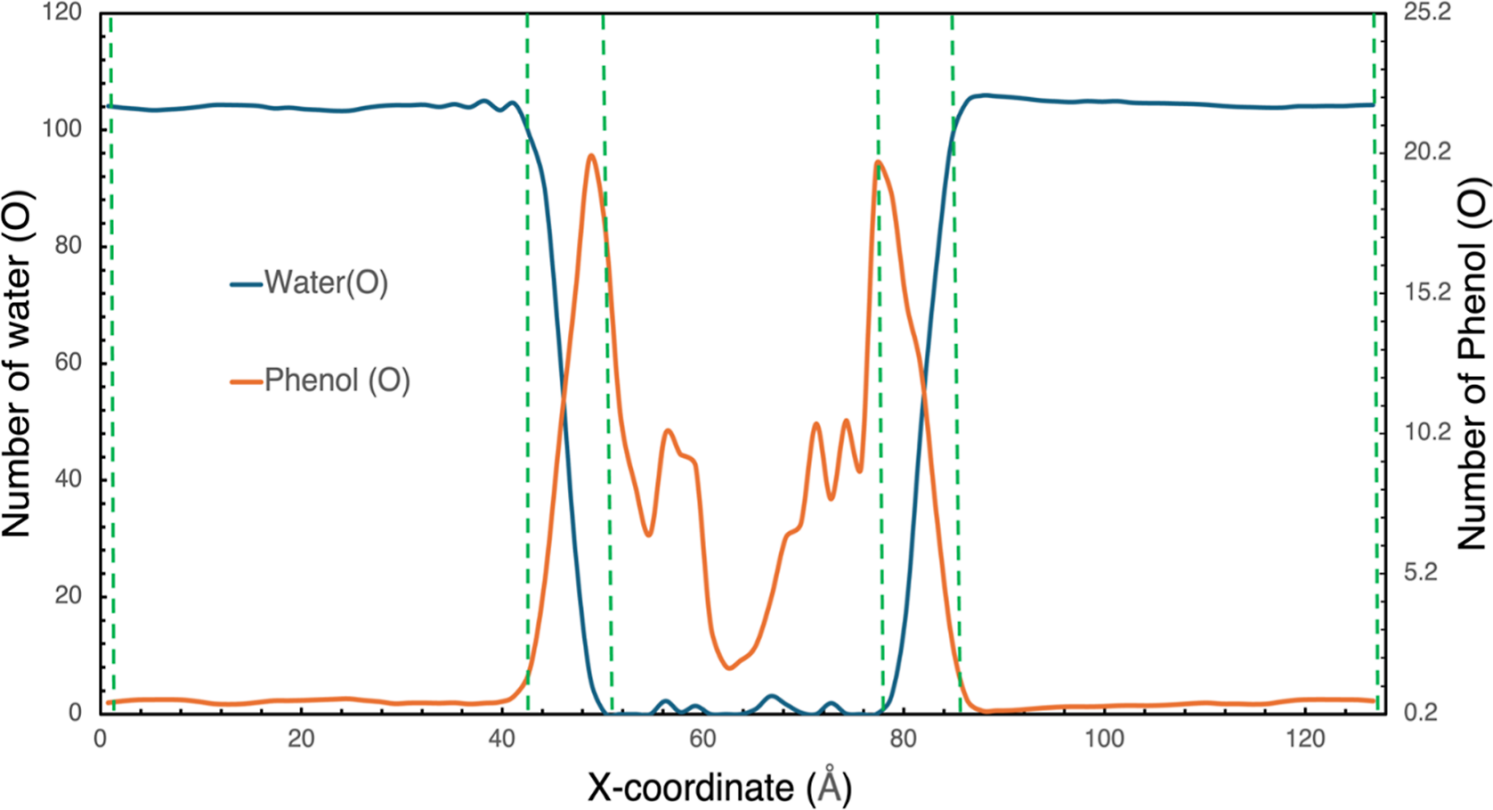
Spatial distribution of water (blue line) and phenol (orange line) molecules along the *x*-coordinate of water-PDMS system. Initially, the PDMS phase is at *x* = 50 Å to 78 Å and the water phases are 0 to 48 Å and 80 to 128 Å.

**Figure 9:**
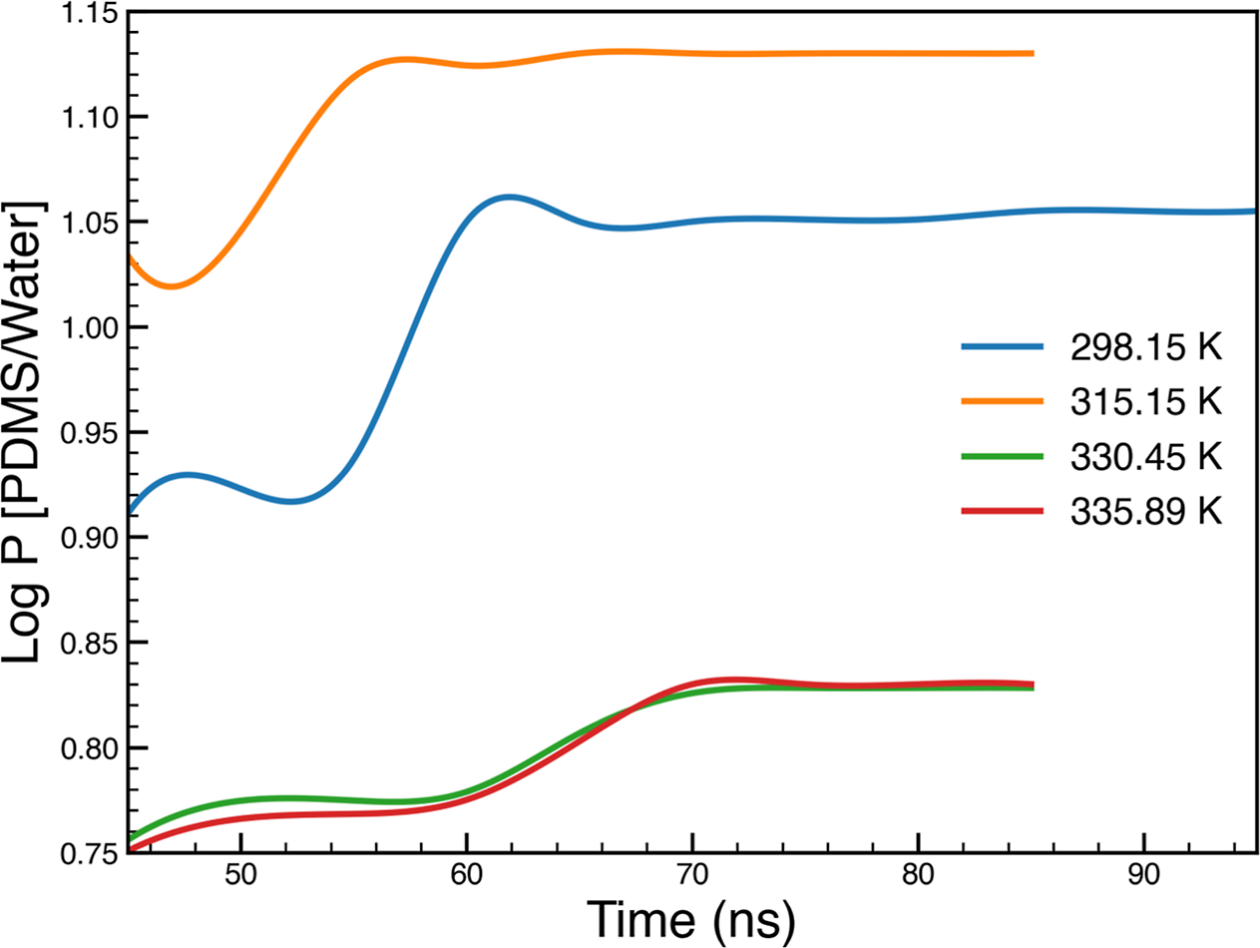
Time evolution of log *P* [PDMS/water] shows approach to equilibrium over time, for phenol, as an example.

Compiling the density profile of the system used a bin size of 1.5 Å, using the coordinates of an atom nearest the center of mass of the molecule. A typical density profile is shown in Figure 8. Molecules within 3 Å of the PDMS interface (dashed vertical green lines) were excluded from the calculation of the concentrations in the two phases to avoid interfacial adsorption effects. In the analysis of the simulations, we used block averages to estimate the precision of our simulations for each organic compound.

## Results and Discussion

To obtain reliable results using MD, the simulations have to be rather long to enable accurate calculations of partitioning and other behavior. Figure 9 shows the log *P* [PDMS/Water] of phenol as a function of the simulation time for each temperature: 298.15, 315.15, 330.48, and 335.89 K. Figure 9 shows that the log *P* values at all temperatures stabilize within the 60–100 ns of simulation time. This method of validation was repeated for all other studied organic compounds: chlorophenol, guaiacol, benzyl alcohol, and phenethyl alcohol, at all temperatures to make sure the result reflects equilibrium conditions. This is important for providing confidence in the distribution, solubility, and diffusion results obtained from the simulations.

The nonmonotonic temperature dependence of the log *P* [PDMS/water] can be explained by the following thermodynamic argument. The solubility is driven by the change in free energy, Δ*A* = Δ*U* – *T*Δ*S*. At room temperature the mixing is endothermic^42^ – implying Δ*U* is positive, but mixing leads to a positive Δ*S*. These competitive driving forces lead to the complex behavior observed for phenol in water compared to phenol in PDMS, where these opposing driving forces are not present. Until about 313 K, the changes in Δ*A* favor phenol in PDMS and the log P [PDMS/Water] as a result increases. Around 335 K the phenol water mixture start essentially forming a single phase (almost complete miscibility) so the Δ*A* now favors the water phase and the log *P* [PDMS/water] starts decreasing.

### Distribution of Molecules into the Two Phases

In Figure 8, the density profiles of the phenol and water in the PDMS system at 298.15 K are reported based on our MD simulations to provide spatial information about the distribution of molecules in the simulation box. Water showed a steep density drop at the boundary of PDMS, indicating very limited penetration into the hydrophobic polymer phase. On the other hand, phenol shows high penetration into the PDMS phase. We also observed density peaks for phenol in the PDMS at the interfacial regions close to 40 and 80 Å along the *x*-coordinate.

Distributions of molecules in water and PDMS phases for each compound obtained from our MD simulation at all temperatures are summarized in Table 3 for partitioning behavior and water content in the PDMS phase. The simulations showed some trends in the preference of phases and molecular distribution. From Table 3 one can clearly see how changing molecular structure and increasing temperature influence the movement of each compound across the water-PDMS interface.

**Table 3:** Distribution of Organic Compounds Across the Water and PDMS Phases at Various Temperatures, Along with the Number of Water Molecules in the PDMS Phase

compound	*T* (K)	number of organics		log *P* [PDMS/water]	water in PDMS phase^a^
		water phase	PDMS phase		
phenol	298.15	50 ± 4	140 ± 1	1.0 ± 0.06	12 (0.1%)
	315.15	41 ± 3	143 ± 2	1.09 ± 0.04	17 (0.3%)
	330.48	73 ± 3	144 ± 2	0.80 ± 0.04	21 ± 1(0.3%)
	335.89	84 ± 4	148 ± 2	0.78 ± 0.05	29 ± 2 (0.5%)
chlorophenol	298.15	22 ± 1	117 ± 1	1.29 ± 0.07	28 (0.4%)
	315.15	66 ± 2	120 ± 1	0.82 ± 0.08	39 ± 2(0.6%)
	330.48	70 ± 1	121 ± 1	0.78 ± 0.095	44 ± 2 (0.7%)
	335.89	65 ± 1	124 ± 1	0.80 ± 0.09	44 ± 2 (0.7%)
guaiacol	298.15	27 ± 1	86 ± 1	1.03 ± 0.05	23 (0.35%)
	315.15	57 ± 1	90 ± 1	0.74 ± 0.04	28 ± 1(0.43%)
	330.48	55 ± 1	96 ± 1	0.77 ± 0.033	31 ± 1(0.48%)
	335.89	48 ± 1	99 ± 1	0.75 ± 0.04	46 ± 2(0.71%)
benzyl alcohol	298.15	33 ± 1	118 ± 1	0.97 ± 0.05	35 (0.55%)
	315.15	48 ± 1	119 ± 1	0.95 ± 0.06	35 ± 1 (0.55%)
	330.48	56 ± 1	124 ± 1	0.90 ± 0.045	38 ± 1(0.59%)
	335.89	68 ± 1	125 ± 1	0.82 ± 0.05	40 ± 1(0.62%)
phenethyl alcohol	298.15	31 ± 1	91 ± 1	0.92 ± 0.06	32 ± 1(0.45%)
	315.15	41 ± 1	98 ± 1	0.93 ± 0.04	38 ± 1 (0.6%)
	330.48	73 ± 1	107 ± 1	0.72 ± 0.05	38 ± 1 (0.6%)
	335.89	69 ± 1	108 ± 1	0.75 ± 0.05	32 ± 1(0.49%)

a Percentages reflect fractions of the total 6418 water molecules in the system.

**Figure 10:**
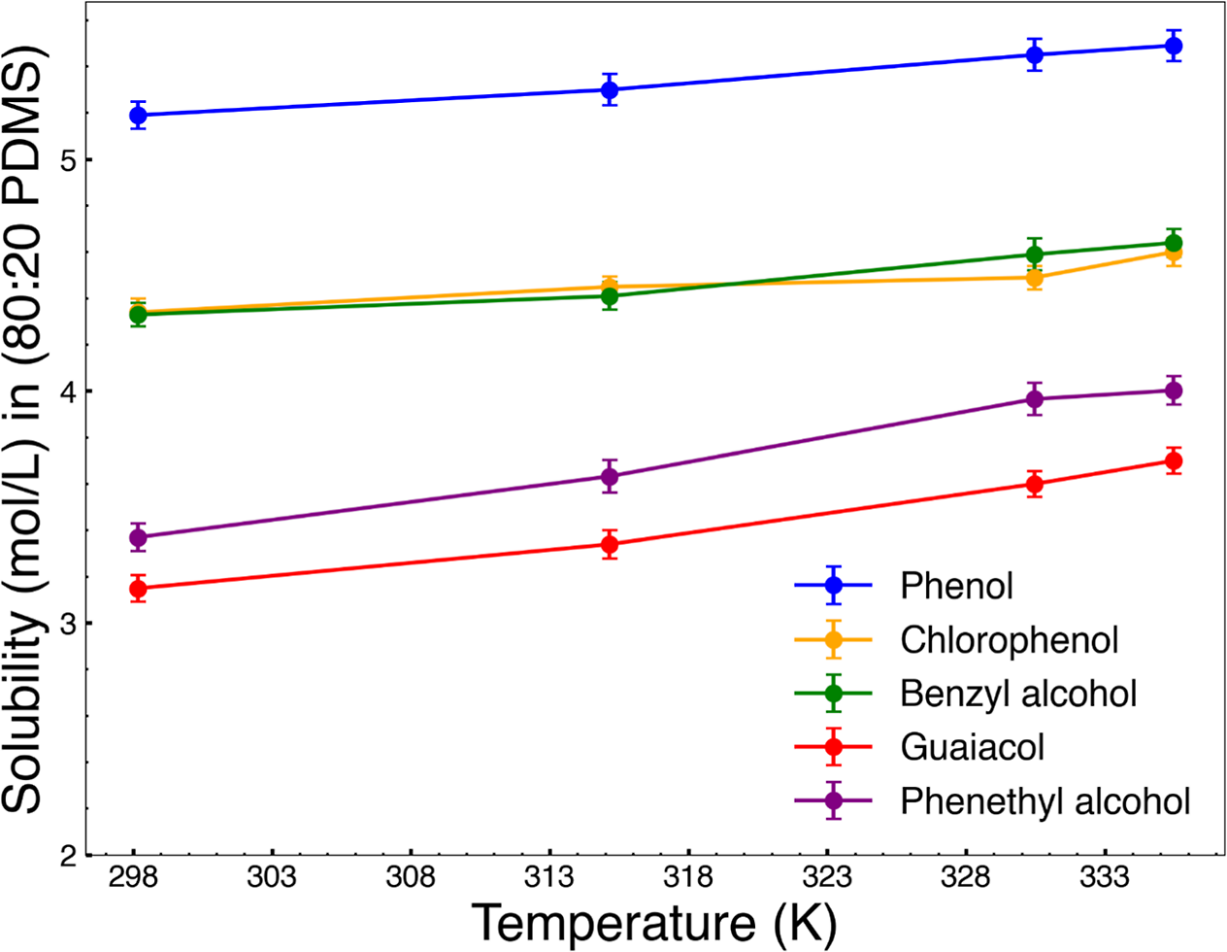
Solubility in the PDMS phase increases with temperature for all compounds, with phenol showing the highest values.

A clear trend is that the number of molecules of the organic compounds in PDMS increases monotonically with increasing temperature for all compounds. In order to supplement the information regarding the compatibility of each compound with the PDMS matrix, we convert these numbers in the fourth column of Table 3 to solubilities (mol/L) in the 80:20 mixture PDMS phase. At 298.15 K solubilities in mol/L are in the following order: 5.19 for phenol, 4.33 for benzyl alcohol, 4.34 for chlorophenol, 3.37 for phenethyl alcohol, and 3.15 for guaiacol. These are shown in Figure 10. Solubility in the PDMS phase generally increases with increasing temperature. Over the temperature range observed here, guaiacol increased by 17.6%, phenethyl alcohol by 7.1%, phenol by 5.7%, chlorophenol by 4.5% and benzyl alcohol by 4.2%.

Phenol values increase from 5.19 mol/L at 298.15 K to 5.49 mol/L at 335.89 K. It is 4.33 mol/L at 298.15 K and 4.64 mol/L at 335.89 K for benzyl alcohol. Chlorophenol solubility, from 4.34 mol/L at 298.15 K up to 4.60 mol/L at 335.89 K. The smallest solubility values were observed for guaiacol, which increased from 3.15 mol/L at 298.15 K to 3.70 mol/L at 335.89 K. Phenethyl alcohol also exhibited low solubility values, increasing from 3.37 mol/L at 298.15 K to 4.0 mol/L at 335.89 K.

### Partition Coefficients

We have shown in Table 2 the solubility of the organic compounds in water. Solubility of the organic compounds in water increases with increasing temperature. The temperature dependence of the solubilities in the aqueous and PDMS phase are similar; both tend to higher solubilities with increasing temperature. But the temperature dependence of the log p[PDMS/water] cannot be predicted from the general trends in solubilities; it was necessary to do the simulations to find how log *P* [PDMS/water] changes with temperature. Partition coefficients log *P* obtained in this work establish the relative affinities of each compound for the PDMS phase vs water; these are summarized in Table 3. At first glance, the values at 298.15 K give the relative order of partition coefficients for the organic compounds in PDMS/water as chlorophenol > guaiacol > phenol > benzyl alcohol > phenethyl alcohol. One can make reasonable explanations for this relative order based on the chemical structures of these molecules. For example, one could note that the chlorine substituent gives enhanced van der Waals interaction with PDMS compared to guaiacol, while guaiacol has the methoxy group substituent, which enhances van der Waals interactions with PDMS relative to the parent phenol compound. However, things are not so straightforward when we look into the temperature dependence of numbers of molecules in each phase. In Table 3, we observe a strong preference for phenol partitioning in the PDMS phase—140 ± 1 molecules in the PDMS phase versus 50 ± 4 molecules in the water phase at 298.15 K. Upon increasing temperature to 335.89 K, the amount of phenol molecules in the PDMS phase increased slightly to 148 ± 2, while 84 ± 4 molecules were present within the water phase. This redistribution suggests that, at higher temperatures, phenol gained increased mobility and, increased their numbers in the PDMS phase, while still more are available in the aqueous phase, despite a smaller log P at the higher temperature. Similarly, chlorophenol partitioned consistently into the PDMS phase throughout the various temperatures studied. At 298.15 K, 117 ± 1 molecules were in the PDMS phase while only 22 ± 1 were in the water phase. This distribution increased at 335.89 K, where 121 ± 1 were in PDMS, and 65 ± 1 were in water. The higher affinity of chlorophenol for the PDMS phase may be a reflection of structural features; the chlorine atom affords greater van der Waals interactions with the PDMS. It was observed that the numbers of benzyl alcohol molecules in the PDMS phase has increased gradually with the increase in temperature: from 117 ± 1 at 298.15 K to 125 ± 1 at 335.89 K. At 298.15 K, there were 31 ± 1 molecules of phenethyl alcohol present in the water phase and 91 ± 1 molecules in the PDMS phase. The number within the PDMS phase increased to 98 ± 1 when the temperature increased to 315.15 K. The number further increased to 107 ± 1 at 330.48 K, while at 335.89 K, 108 ± 1 phenethyl alcohol molecules remained in the PDMS phase. These results confirm that upon raising the temperature, the number of phenethyl alcohol increases within the PDMS phase, exhibiting a behavior similar to that of guaiacol. These findings account for the critical interaction between the molecular structure and temperature as the key determinants of the partitioning behavior of the organic compounds between the water and PDMS phase.

**Figure 11:**
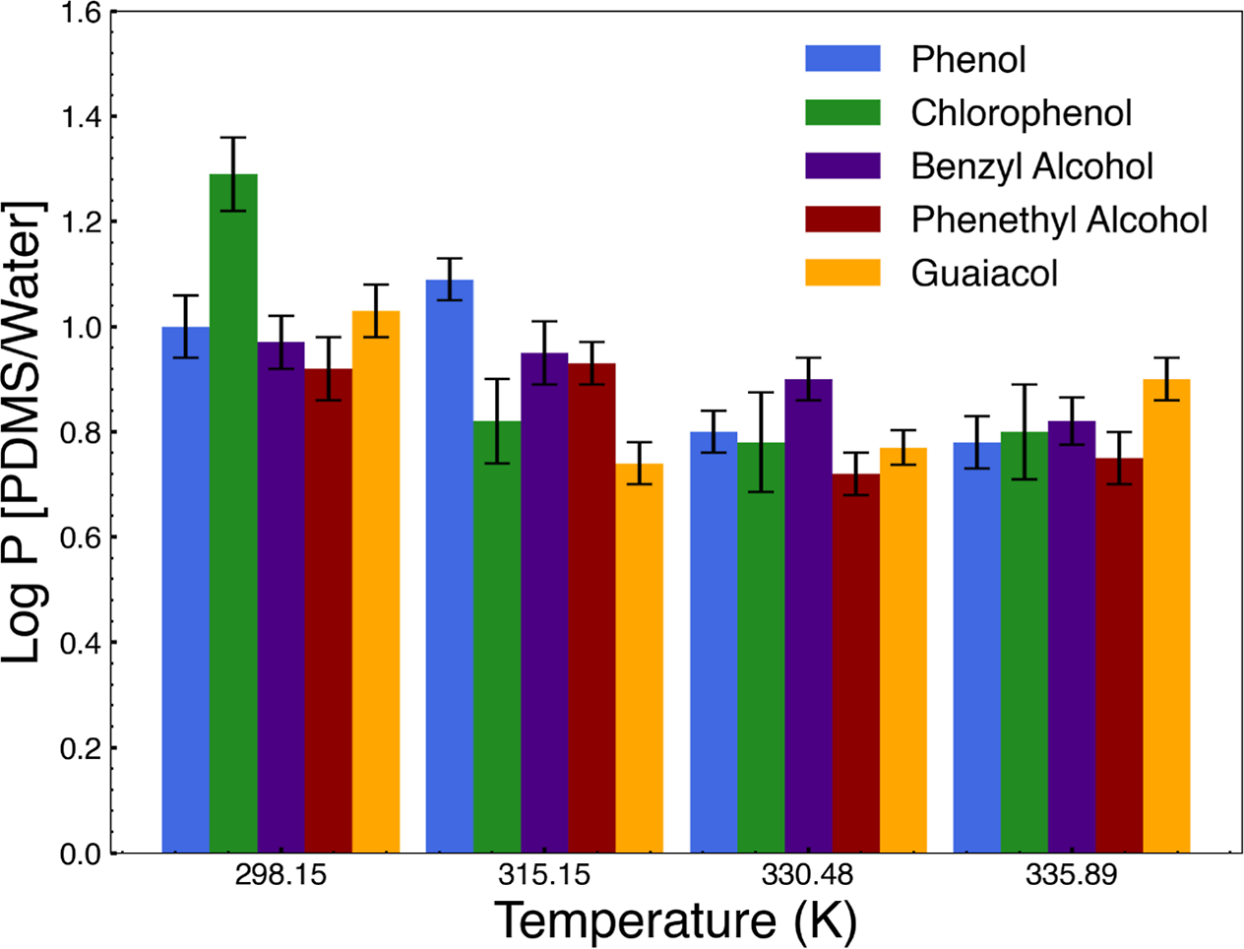
Temperature dependence of log P[PDMS/water].

However, the temperature dependence of log P[PDMS/water] is not straightforward to predict from structural arguments, as can be seen in Figure 11. For instance, in the case of phenol, there was a dramatic decrease in log *P* values with an increase in temperature from 1.0 ± 0.06 at 298.15 K to 0.78 ± 0.05 at 335.89 K. The log *P* obtained with chlorophenol was the highest: 1.29 ± 0.07 at 298.15 K, which indicated a stronger affinity for the PDMS phase. This value had decreased to 0.80 ± 0.09 by 335.89 K but was still higher than the log *P* values of phenol at the same temperature. The log *P* values obtained for benzyl alcohol showed a very slight decrease from 0.88 ± 0.05 at 298.15 K to 0.82 ± 0.04 at 335.89 K. Such stability in log *P* values could be indicative of a weaker dependence on temperature. In the case of phenethyl alcohol, the log *P* values range from 0.89 ± 0.07 at 298.15 K to 0.84 ± 0.08 at 335.89 K, reflecting weaker affinity for the PDMS phase compared with the other tested compounds. For guaiacol, the log *P* values were highest at 1.10 ± 0.08 at 298.15 K, denoting a very strong affinity for the PDMS phase at room temperature. The log *P* values decrease with temperature only moderately to reach 0.90 ± 0.07 at 335.89 K but resulted in a rather high retention in the PDMS phase when compared to the other compounds.

Another interesting observation is the amount of water within the PDMS phase. The solubility of water in PDMS is rather small. In nearly all cases, the mol % of water in the PDMS phase increases monotonically with increasing temperature, except in the extraction of phenethyl alcohol where it goes from 0.45, 0.6, 0.6, and down again to 0.49 mol %. From the above results and log *P* values it can be said that although the partitioning of phenethyl alcohol into the PDMS phase is poorer compared to the other tested compounds at room temperature, the nature of the interaction of water with the PDMS phase changes subtly with the change in temperature due to differential changes in the polymer free volume in the presence of molecular interactions.

### Diffusion Coefficients of Organics in PDMS

**Figure 12:**
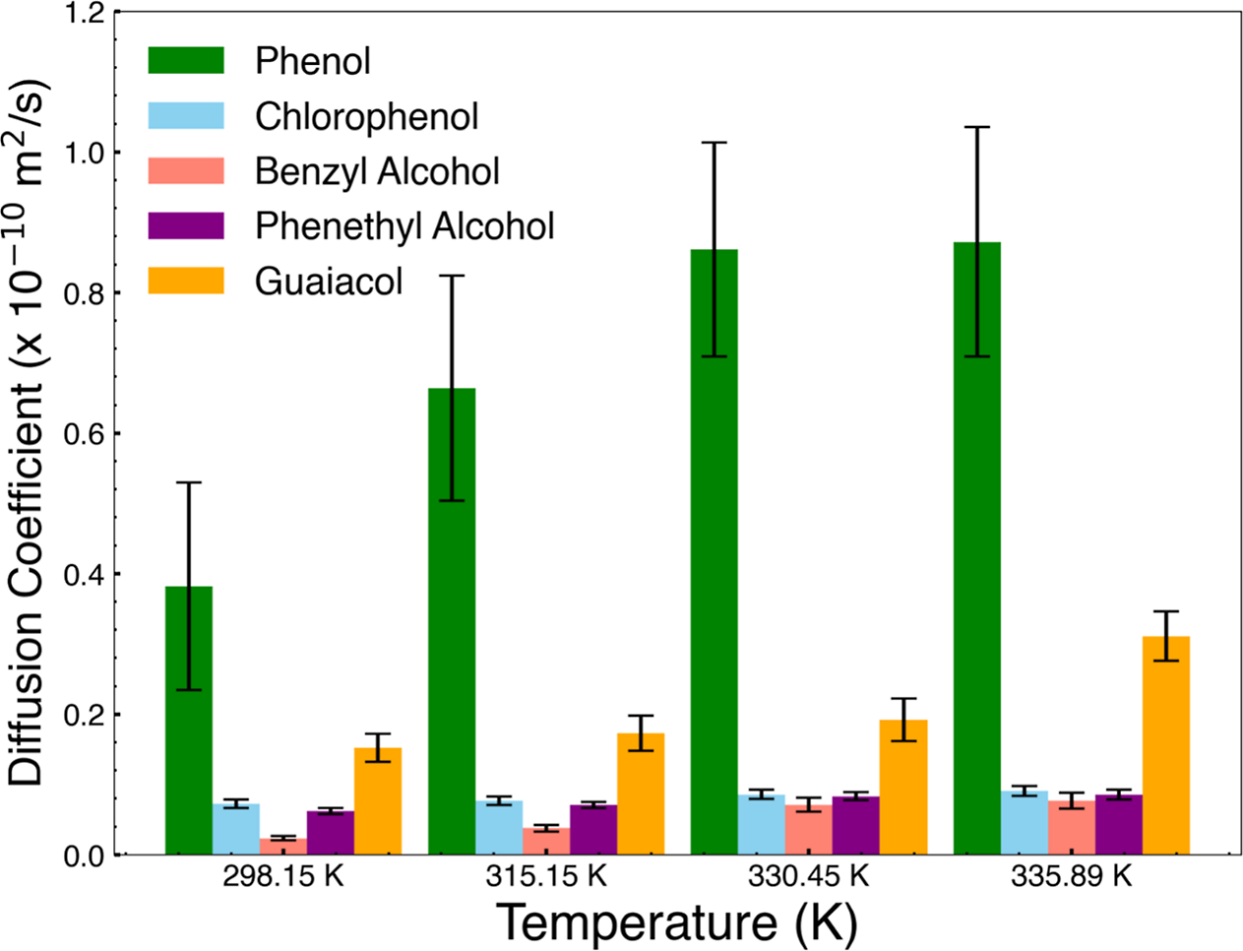
Diffusion coefficients in the PDMS phase.

We have used the diffusion coefficient of phenol in aqueous solution to validate our force field parameters because experimental values are available and also extend the data to higher temperatures after validation. As a measure of the ease of penetration of the compounds into the PDMS phase, we calculated the diffusion coefficients of phenol, chlorophenol, guaiacol, benzyl alcohol, and phenethyl alcohol in the PDMS phase from 298.15 K up to 335.89 K. Results are shown in Figure 12. Diffusion coefficients in the PDMS phase show phenol diffuses fastest, increasing sharply with temperature. Chlorophenol showed relatively lower values of the diffusion coefficient, its increase with a rise in temperature being moderate. For benzyl alcohol, the diffusion coefficients were even lower compared to chlorophenol, but their increase with the rise in temperature was stronger. For phenethyl alcohol, the diffusion coefficients were the lowest among the compounds under investigation; the increase with the rise of temperature was minor. On the other hand, guaiacol presented diffusion coefficients higher than those of chlorophenol and benzyl alcohol, with greater increases with the rise in temperature. These data illustrate the dependence of molecular mobility on temperature for the organic compounds studied in an 80:20 PDMS mixture phase.

Even though the adsorption of the organic molecules at the PDMS surface is an important first step toward its incorporation into the bulk PDMS, this is only a dynamic effect. We carry out long enough simulations so that this dynamic effect only affects the kinetics of the approach to equilibrium. In fact, the regions at the interfaces between water and PDMS phases (indicated by dashed vertical lines in Figure 8) are excluded from the calculations of average density in the individual phases. Our primary interest is the equilibrium property, the partition coefficient.

Equilibration of MD simulations is a very important step that guarantees the properties calculated, such as logP [PDMS/water] values and the behaviors observed, are truly representative of near-equilibrium states rather than transient results. The long simulation times used in this work highlight the need for sufficient time scales in order to approach equilibrium in complex systems. Our simulations indicate that simulation times of the order of 60–100 ns are necessary to stabilize phase partitioning between the polymer and the surrounding aqueous environment. This requirement is especially relevant for phase partitioning investigations in polymer matrices, where the dynamics are intrinsically slower due to the presence of several molecular interactions in the system, such as the van der Waals forces and hydrophobic effects.

Focusing on the individual compounds, distinct tendencies can be observed for the partitioning behavior of phenol, chlorophenol, benzyl alcohol, phenethyl alcohol, and guaiacol with respect to molecular structure and temperature. We chose two families with benzene rings: phenolic compounds phenol, chlorophenol, and guaiacol and alcohols benzyl alcohol and phenethyl alcohol.

The general increase in solubility with increasing temperature of the organic compounds in the PDMS phase that has been demonstrated here can be accounted for by the underlying mechanisms of thermal stretching of the PDMS phase and the associated generation of transient free volumes.^43^ However, the relative order of the increase in solubility with 15.1% for guaiacol as the highest and 5.9% for benzyl alcohol as the smallest increase is not straightforward to explain. These characteristics favor molecular diffusion and allow for the accommodation of more organic molecules. Whereas theoretically, hydrogen bonding interactions of the OH group in the phenols and the alcohols with the siloxane backbone are possible, partitioning is mainly determined by van der Waals interactions and the hydrophobic effect rather than hydrogen bonding. This is in good agreement with the general observation that polar molecules do not show high affinity for PDMS-coated stirrer bar without derivatization.^24^ The partition coefficient log P[PDMS/water] determines the ratio of a compound’s concentration in the PDMS phase to its concentration in the aqueous phase. The solubility of the organic compounds in water and in PDMS both tend to increase with increasing temperature. Thus, the log P temperature dependence will depend on the relative values of the temperature coefficients of the solubilities in the two phases. The thermal expansion of PDMS at elevated temperatures creates transient free volumes within the polymer matrix, facilitating the mobility of analytes and water molecules while maintaining hydrophobic exclusion of water. In the MD simulations for the sorption studies, the increased mobility of the PDMS chains are properly described; in addition, the expansion of the PDMS phase with increasing temperature is also properly described because the PDMS is put through simulated annealing for each temperature prior to the setup of the simulation box. This thermal response of PDMS is critical in explaining the temperature-dependent trends observed in our simulations. We find that the partition coefficient does not vary with temperature in the same way for the different compounds. The relative order of log *P* values at room temperature is not preserved when temperature is increased, as seen in Figure 11. Benzyl alcohol and phenethyl alcohol behave analogously; increasing temperature appears to have diminished the dynamic interactions of such organic molecules with the PDMS phase and increased their desorption from PDMS toward the aqueous phase. This temperature-induced desorption is consistent with increased molecular mobility and weakening of intermolecular forces, a behavior very well explained in the literature through several cases dealing with polymer sorption systems.^44^ At 298.15 K, a higher partitioning of chlorophenol compared to phenol could be attributed to the chlorine substituent intensifying van der Waals interactions with the PDMS; hence, increasing the log P compared to phenol. This behavior is consistent with experimental results indicating that chlorophenol demonstrates consistently superior extraction efficiencies relative to phenol, highlighting the importance of hydrophobicity and molecular architecture in influencing partitioning characteristics.^20,45^ However, the relative order of log *P* for these two compounds is reversed at 315 K.

Whereas the qualitative trend observed in this work is a decrease of log *P* with the increase in temperature, the total number of organic molecules that partition into the PDMS phase, in all cases increased with temperature because of increased molecular diffusion and solubility. This is a key feature for completeness of extraction whether the compound being extracted is an undesirable toxic contaminant or is a valuable desired component. These findings corroborate the ability of molecular dynamics simulations to accurately represent temperature-dependent sorption patterns and offer molecular-level understanding of the interactions that dictate the partitioning of phenolic compounds between polydimethylsiloxane and water. These results also demonstrate how robust PDMS is as a sorptive material in SBSE, maintaining its efficiency as a selective absorber under conditions of competitive sorption with water even at elevated temperatures.

Besides the partitioning tendencies observed, the concentration of the water molecules retained in the PDMS phase also gives additional insight into the mechanism of hydrophobic exclusion and temperature-induced permeation. From Figure 8 which illustrates the distribution of molecules in the water-PDMS system through the density profile of phenol and water, the sharp decline of water density at the interface with PDMS confirms one of the important characteristics of PDMS: it is hydrophobic, hence excludes water molecules. Table 3 shows that during all simulations, less than 1% of the 6418 water molecules present in the system are found within the PDMS phase, an indication of its strong hydrophobic nature. The minimum absorption of water, at 298.15 K, reveals the high resistance of the polymer toward the penetration of water, with values lying between 0.1 and 0.7 mol %, depending on the organic molecule in the tricomponent phase. The higher value of water content at higher temperatures (up to 0.7 mol %) reflects an increase in molecular mobility and thermal agitation, yet this is still of a small magnitude. Out of the transient free volumes formed by thermally induced dilation of the polymer matrix, only a small fraction is utilized by water for diffusive transport. Consistent with its robust hydrophobic exclusion properties, PDMS resists water penetration well, demonstrating its effectiveness as a selective sorptive material in SBSE applications, predominantly accommodating organic molecules while minimizing absorption of water, even under variable thermal conditions.

The diffusion coefficients of phenol, chlorophenol, benzyl alcohol, phenethyl alcohol, and guaiacol within the water-PDMS system affect how fast is the extraction using the PDMS stirrer bar. We observed the highest diffusion coefficients in the water-PDMS system by the phenol molecule, and much higher upon increasing the temperature. Higher temperatures reduce viscous resistance of the polymer substantially; hence, the mobility of phenol in PDMS is increased considerably. On the contrary, chlorophenol exhibited much lower diffusion coefficients with a moderate increase upon rising temperature. With the chlorine substituent on the ring, the molecular mobility of chlorophenol is reduced compared to that for phenol due to its larger size and larger van der Waals interactions with the PDMS phase. Of all the compounds studied, the lowest diffusivities obtained were for phenethyl alcohol and this increased negligibly with an increase in temperature. The much larger molecular size and polar hydroxyl group is highly significant in reducing its diffusivity in the PDMS phase. Hence, it turns out to be the least mobile compound in this system, even at higher temperatures. Guaiacol presents an intermediate trend for diffusivity, with coefficients above those of chlorophenol and benzyl alcohol, increasing considerably with a rise in temperature. Favorable van der Waals interactions with the PDMS matrix are provided by the methoxy group (−OCH_3_) on the ring compared to phenol. A balance of polarity and hydrophobicity allowed better diffusivity compared to benzyl alcohol. All compounds studied showed an increased diffusion coefficient with a rise in temperature; thermal energy increases the mobility of the molecules within the PDMS phase. The degree of increase naturally depends on other parameters such as molecular size, polarity, and an attached functional group on the diffusing compound. The smaller, less polar phenol showed a high diffusion coefficient, whereas the larger chlorophenol (relative to phenol) and phenethyl alcohol (relative to benzyl alcohol) faced strong resistance. The intermediate behavior of guaiacol underlined the importance of the balance between functional group contributions to polarity and van der Waals interactions. Such findings have provided a molecular level insight into the temperature-driven diffusion process in PDMS, and the ability of PDMS to selectively accommodate organic molecules in SBSE applications. Trends in diffusion coefficients are consistent with those of solubility and stress the importance of molecular architecture and temperature in determining the nature of sorption and transport of organic analytes within the PDMS phase.

## Conclusions

In the present work, the behavior of representative compounds of two chemical families phenols and benzyl alcohols, specifically phenol, chlorophenol, and guaiacol of the former and benzyl alcohol and phenethyl alcohol of the latter, has been investigated in both water and PDMS phases within the temperature range from 298.15 K up to 335.89 K. Distribution of the molecules, partition coefficient log P [PDMS/water], trends of solubility, amounts of water in the PDMS phase, system density profiles, and diffusion coefficients were obtained in molecular dynamics simulations but required long simulation times before the partition coefficients leveled off. MD simulations provide the actual partition coefficients rather than the usual surrogate quantities estimated from octanol–water partition coefficients. The temperature dependence of these quantities was found to be consistent with the nature of the van der Waals and hydrogen bonding interactions between the molecules and the solvents water and PDMS. Our results obtained in this work provide complete information on the diffusive behavior of essential organic species in PDMS and simultaneously give insight into the dependence of the extraction process on temperature. Although there is a qualitative trend of decreasing log P [PDMS/water] values, the increase in the absolute number of organic molecules-phenol, chlorophenol, guaiacol, benzyl alcohol, and phenethyl alcohol-in the PDMS phase with the rise in temperature justifies performing SBSE at high temperatures to achieve high sorption capacity. Such an increase is related to the enhanced molecular diffusion under these kinetic-driven conditions, coupled with a higher sorption capacity of PDMS that can accommodate more organic molecules at the higher temperature. Our results suggest that performing SBSE at higher temperatures has the advantage of enhancing the sensitivity and efficiency of the analytical measurements, especially for target analytes of intermediate hydrophobicity. Thus, better enrichment factors of trace organics or more complete removal of undesirable contaminants in water can be achieved, not only in analytical, but also in environmental contexts. This information is of utmost importance in designing an efficient extraction protocol and optimizing the performance of SBSE. The results obtained here form a basis for further studies that may focus on the variation of sorptive and diffusive properties subsequent to structural changes in PDMS, such as cross-linking or blending. Experimental verification of the computed diffusion coefficients of organic compounds within the PDMS phase, using techniques like PFG-NMR, would enhance the reliability of the computational predictions and give a vision of the important role that MD simulations are going to play in the development of an understanding of interfacial separation techniques like SBSE.
